# Periodicity and positivity of a class of fractional differential equations

**DOI:** 10.1186/s40064-016-2386-z

**Published:** 2016-06-22

**Authors:** Rabha W. Ibrahim, M. Z. Ahmad, M. Jasim Mohammed

**Affiliations:** Faculty of Computer Science and Information Technology, University of Malaya, 50603 Kuala Lumpur, Malaysia; Institute of Engineering Mathematics, Universiti Malaysia Perlis, 02600 Arau, Perlis Malaysia

**Keywords:** Fractional calculus, Fractional differential equations, Rayleigh equation

## Abstract

Fractional differential equations have been discussed in this study. We utilize the Riemann–Liouville fractional calculus to implement it within the generalization of the well known class of differential equations. The Rayleigh differential equation has been generalized of fractional second order. The existence of periodic and positive outcome is established in a new method. The solution is described in a fractional periodic Sobolev space. Positivity of outcomes is considered under certain requirements. We develop and extend some recent works. An example is constructed.

## Background

Fractional calculus is the important branch of mathematical analysis field, it covenants with the requests and exploration of derivatives and integrals of random order. The fractional calculus is deliberated an old and yet original study. It has been planned a long time ago, beginning from some conjectures of Leibniz (1695, 1697) and Euler (1730), selected investigators have been established it up to nowadays. In latest years, fractional calculus has been encouraged through the presentations that discovers in numerical analysis and diverse grounds of engineering and physics, possibly including fractal phenomena (Gorenflo and Mainardi 2008).

Fractional calculus has been developed significantly within the historical three decades for the reason that its applicability in various branches of science, engineering and social. The philosophies of fractional calculus may be sketched back to the Euler’s works of, but the indication of fractional difference is very currently (Yang 2012). At the present time, a mounting number of effort by many investigators from various fields of engineering and science deal with dynamical systems designated by fractional partial differential equations. Outstanding to the extensive applications of Fractional differential equations (FDEs) in engineering and science, this capacity of investigation has developed meaningfully all around the world (Yang 2015).

Fractional differential equations (FDEs) are generalizations of the class of ordinary differential equations (ODEs) for a random (non-integer) order. FDEs have expanded desirability and substantial interests due to their ability to simulate a complex phenomena. These equations capture nonlocal relations in space and time with power-law memory kernels (Liu 2010). In this effort, we shall deal with (generalize and extend) special class of differential equations of arbitrary order. The British mathematical physicist Lord Rayleigh introduced an equation of the form (Strutt 1877)

$$\begin{aligned} x''(t)+f( x'(t) )+ax(t)=0 \end{aligned}$$

to model a clarinet reed oscillation; which is showed by Wang and Zhang (2009). This equation was named after Lord Rayleigh, who studied equations of this type in relation to problems in acoustics. In the years of 1977 and 1985 respectively, Gaines and Mawhin (1977) have been imposed some continuation theorems and employed them to demonstrate the existence of periodic solutions to ordinary differential equations (ODEs). A specific example was given in Yang (2015, p. 99) to introduce, how *T*-periodic solutions can be obtained by using the established theorems for the differential equation of the form

$$\begin{aligned} x'' (t)+f(x'(t) )+g(t,x(t))=0. \end{aligned}$$

Newly, investigators discussed the existence of periodic solutions to Rayleigh equations and extended Rayleigh equations by considering or ignoring the concept of delay. Various new results concerning the existence of periodic solutions to the mentioned equations have been presented.

Wang and Yan (2000) established the existence of periodic solutions of the non-autonomous Rayleigh equation of the type:

$$\begin{aligned} x'' (t)+f(t,x'(t-\tau ) )+g(t,x(t-\sigma ))=p(t). \end{aligned}$$

Zhou and Tang (2007) have studied the existence of periodic solutions for a kind of non-autonomous Rayleigh equations of retarded type:

$$\begin{aligned} x'' (t)+f(t,x'(t-\sigma ) )+g(t-\tau (t))=p(t). \end{aligned}$$

Wang and Zhang (2009) investigated the following Rayleigh type equation:

$$\begin{aligned} x'' (t)+f(x'(t) )+g(t,x(t))=e(t). \end{aligned}$$

In this study, we consider a Rayleigh-type equation with state-dependent delay of the form

1 $$\begin{aligned} D^{2\mu }u(t)+\Psi (u(t))D^{\mu }u(t)+\varphi (t,D^{\mu }u(t-\varpi ))+\vartheta (t,u(t-\varepsilon (t,u(t))))=p(t) \end{aligned}$$

and its conducive formal

2 $$\begin{aligned} D^{2\mu }u(t)+\omega \Psi (u(t))D^{\mu }u(t)+\omega \varphi (t,D^{\mu }u(t-\varpi ))+\omega \vartheta (t,u(t-\varepsilon (t,u(t)))) =\omega p(t), \end{aligned}$$

where

$$\begin{aligned} \Big ( \omega \in (0,1),\varpi \ge 0,\varphi , \vartheta \in C(\mathbb {R}^{2},\mathbb {R}),\varepsilon \in C (\mathbb {R}^{2},\mathbb {R}^{+}), \mathbb {R}^{+}=[0,\infty ) \Big ), \end{aligned}$$

$\varphi$ and $\vartheta$ are $2\pi$-periodic in $t,\varphi (t,0)=\vartheta (t,0)= 0$ for $t \in \mathbb {R} , \Psi , p \in C(\mathbb {R},\mathbb {R}),\varepsilon , \, p$ are $2\pi$-periodic in *t*, such that *p* has the property:

$$\begin{aligned} \int ^{2\pi }_{0}p(t)= 0 \end{aligned}$$

and $D^{\mu }$ is the Riemann–Liouville fractional differential operator.

We have imposed two contributed theorems on the existence of periodic solutions of Eq. (1). Our main aim is to generalize, modify and extend the outcomes of the works given in Wang and Yan (2000), Zhou and Tang (2007), Tunç (2014). In addition, this effort is a contribution to the subject in the literature and it may be useful for researchers who work on the qualitative behaviors of solutions. Positivity of solutions is investigated under some requests. Our method is based on the idea of the continuation partition theorem of degree theory. Applications are illustrated in the sequel.

## Setting

In this paper, we need the following setting. For the sake of convenience, let

$$\begin{aligned} C_{2\pi }=\lbrace { u : u \in C (J , \mathbb {R}), u(t + 2\pi ) = u(t), t \in J:=[0,2 \pi ] }\rbrace , \end{aligned}$$

that is, the Sobolev space $W^{k,p}(J)$ is defined as

$$\begin{aligned} W^{k,p}(J) = \left\{ u \in L^p(J) : D^{\mu }u \in L^p(J) \,\, \forall |\mu | \leqslant k \right\} , \end{aligned}$$

with the order of the Sobolev space $(W^{k,p}(J)) k \in \mathbb {N}.$

$$\begin{aligned} \Vert u \Vert _{0}= & {} \max _{t\in [0,2\pi ]} \vert u(t)\vert< \infty ,\\ \Vert D^{\mu } u \Vert _{0}= & {} \max _{t\in [0,2\pi ]} \vert D^{\mu } u(t)\vert <\infty . \end{aligned}$$

In the sequel, we assume that $k=1.$ Hence, we deal with the fractional periodic Sobolev space of a continuous integrable function $u(t), \, t \in [0,2 \pi ]$

$$\begin{aligned} W^{1,p}([0,2 \pi ]) = \left\{ u \in L^p([0,2\pi ])\cap C_{2\pi } : D^{\mu }u \in L^p([0,2\pi ]) \,\, \forall \mu \leqslant 1 \right\} . \end{aligned}$$

Note that the above space is formulated as a Banach space.

### **Definition 1**

Let $\mathcal {X}, \Vert .\Vert$ be a Banach space. Then $\phi : \mathbb {R} \rightarrow \mathcal {X}$ is called periodic if $\phi$ is continuous, and for each $\varepsilon {>} 0$ such that for a number *t* with the property that

$$\begin{aligned} \Vert \phi (t+\tau )-\, \phi (t)\Vert < \varepsilon , \end{aligned}$$

for each $$t \in \mathbb {R}.$$

Fractional order integral and differentiation were obtained by Leibniz. To analyze phenomena having singularities of type $t^{\mu }$, the concept of fractional calculus is utilized. The fractional order operator is a nonlocal operator. Due to this property, fractional calculus is employed to study memories of Brownian motion, which is thought to be beneficial in mathematical sciences.

### **Definition 2**

The Riemann–Liouville fractional integral defined as follows:

$$\begin{aligned} I^{\mu } u(t) = \frac{1}{\Gamma (\mu )} \int ^{t} _{0} (t-\delta )^{\mu -1} u(\delta ) d\delta , \end{aligned}$$

where $\Gamma$ denotes the Gamma function (see Podlubny 1999; Kilbas et al. 2006; Tarasov 2010).

### **Definition 3**

The Riemann–Liouville fractional derivative defined as follows:

$$\begin{aligned} D^{\mu } u(t) = \frac{1}{\Gamma (1-\mu )} \frac{d}{d t }\int ^{t} _{0} (t-\delta )^{-\mu } u(\delta ) d\delta , \quad 0< t< \infty . \end{aligned}$$

The periodicity of the class of fractional differential equations is studied in various spaces, Agarwal et al. studied the periodicity of various classes of fractional differential equations by assuming the mild solution (Agarwal et al. 2010), Ibrahim and Jahangiri (2015) imposed a periodicity method by applying some special transforms for fractional differential equations, and recently, Rakkiyappan et al. (2016) introduced the periodicity by utilizing fractional neural network model. Extra studies in fractional calculus can be located in Khorshidi et al. (2015).

## Results

The subsequent Lemma plays a key function for showing the periodicity of Eq. (1). In the sequel, we assume that $u \in \mathbf {W}:= W^{1,p}([0,2 \pi ]).$

### **Lemma 1**

*Suppose thatu*(*t*) *is a continuous and differentiableT*-*periodic function with*$T<\infty .$*Then there exists*$t_\bullet \in [0,T]$*such that*

3 $$\begin{aligned} \max _{t \in [t_\bullet , t_\bullet + T ]} \vert u(t) \vert \le \vert u(t_{\bullet })\vert + \frac{1}{2} \int ^{T}_{0}\vert D^{\mu } u(\delta )\vert d\delta . \end{aligned}$$

### *Proof*

Let $\overline{t} \in [ t_{\bullet } , t_{\bullet } + T ]$ such that $u(\overline{t} ) = max_{t \in [t_\bullet , t_\bullet + T ]} \vert u(t) \vert .$ Then

$$\begin{aligned} \vert u (\overline{t}) \vert = \vert u (t_{\bullet } ) + \int ^{\overline{t}} _{t_{\bullet }} D^{\mu } u(\delta ) d\delta \vert \le \vert u (t_{\bullet } )\vert + \int ^{\overline{t}}_{t_{\bullet }} \vert D^{\mu } u (\delta ) \vert d \delta \end{aligned}$$

together with the estimate

$$\begin{aligned} \vert u (\overline{t}) \vert =\vert u (\overline{t} -T) \vert = \vert u (t_{\bullet } ) - \int ^{t_{\bullet }} _{\overline{t} -T} D^{\mu } u(\delta ) d\delta \vert \le \vert u (t_{\bullet } )\vert + \int ^{t_{\bullet } }_{\overline{t}-T} \vert D^{\mu } u (\delta ) \vert d \delta . \end{aligned}$$

From the above two inequalities and the Definition 3., we obtain that

$$\begin{aligned} \vert u (\overline{t}) \vert \le \vert u (t_{\bullet } ) +\frac{1}{2} \int ^{\overline{t}} _{\overline{t} -T}\vert D^{\mu } u(\delta )\vert d\delta = \vert u (t_{\bullet } )\vert + \frac{1}{2} \int ^{T}_{0} \vert D^{\mu } u (\delta ) \vert d \delta . \end{aligned}$$

Hence, we complete the proof.

Lemma 1 shows the boundedness of the fractional differential operator by the norm of fractional space. This result allows us to investigate the periodicity of the solutions. If the differential equation satisfies the initial condition $u(t_\bullet )=0,$ then, we can attain

$$\begin{aligned} \int ^{T}_{0} \vert D^{\mu } u (\delta ) \vert d \delta \le \Vert u\Vert . \end{aligned}$$

We have the following main results:

### **Theorem 1**

*Suppose that there exist constants, with the validity*

$$\begin{aligned} \Big (\Upsilon _{1} {>} 0, \,\, \eta _{1}, \, \eta _{2} \ge 0, d {>} 0, \, \kappa {>} 0 \quad \text {and} \quad m {>} 0\Big ) \end{aligned}$$

*such that the following conditions hold:*

1. $$\,\vert \Psi (u) \vert \le \Upsilon _{1}$$ for all $$u \in \mathbb {R};$$
2.   $$\vert \varphi (t,u) \vert \le \Upsilon _{1} \vert u \vert + \kappa$$ for all $$(t ,u) \in J\times \mathbb {R};$$
3.
  $$u \vartheta (t ,u (t-\varepsilon (t,u ))) {>} 0;$$
    $$\vert \vartheta (t , u (t - \varepsilon (t, u)))\vert {>} \eta _{1} \vert u \vert + \kappa$$ for all $$t \in J , \vert u \vert {>} d ;$$
4.   $$\vartheta (t ,u (t-\varepsilon (t,u ))) {>} \eta _{2} u - m$$ for all $$t \in J, u \le -d .$$

*If*

$$\begin{aligned} 2 \pi \lbrace (\Upsilon _{1} + \eta _{1} ) + (\pi + 1 ) \eta _{2} \rbrace < 1 , \end{aligned}$$

*then Eq.* (1) *has at least one*$2 \pi$-*periodic solution.*

### *Proof*

We reconsider the auxiliary equation, Eq. (2). Let $u(t)\in \mathbf {W}$ be any $2 \pi -$periodic solution of Eq. (2). Then, integrating both sides of Eq. (2) from 0 to $2 \pi$, we get

4 $$\begin{aligned}&\int ^{2{\pi }}_0\Psi (u(\delta ))D^{\mu }u(\delta )d\delta +\int ^{2{\pi }}_0{\varphi (\delta ,D^{\mu }u(\delta -\varpi ))+\vartheta (\delta ,u(\delta - \varepsilon (\delta ,u(\delta ))))}d\delta =0\nonumber \\&\quad \Longrightarrow \int ^{u(2{\pi })}_0\Psi (v)dv+\int ^{2{\pi }}_0{\varphi (\delta ,D^{\mu }u(\delta -\varpi ))+\vartheta (\delta ,u(\delta -\varepsilon (\delta ,u(\delta ))))}d\delta =0\nonumber \\&\quad \Longrightarrow \int ^{2{\pi }}_0{\varphi (\delta ,D^{\mu } u(\delta -\varpi ))+\vartheta (\delta ,u(\delta -\varepsilon (\delta ,u(\delta ))))}d\delta =0. \end{aligned}$$

Thus, it yields that there exists a value $t_1\in [ 0,2 \pi ]$ such that

5 $$\begin{aligned} \varphi (t_{1},D^{\mu }u(t_{1}-\varpi ))+\vartheta (t_{1},u(t_{1}-\varepsilon (t_{1},u(t_{1}))))=0 . \end{aligned}$$

We admit that there exists a $\overline{t} \in [ 0,2 \pi ]$ such that

$$\begin{aligned} \vert u(\overline{t})\vert \le \Vert D^{\mu }u\Vert _{0} +d. \end{aligned}$$

**Case 1.** Let $\eta _{1} = 0$. Then

$$\begin{aligned} \vert \varphi (t , u ) \vert \le \eta _{1} \vert u \vert + \kappa \Rightarrow \vert \varphi ( t, u ) \vert \le \kappa . \end{aligned}$$

From the last conclusion and (5), we obtain

$$\begin{aligned} \vert \vartheta ( t_{1} , u (t_{1} - \varepsilon (t_{1} , u (t_{1} ))) \vert \le \kappa . \end{aligned}$$

The last assertion alongside with the hypotheses

$$\begin{aligned} \vert \vartheta ( t_{1} , u (t_{1} - \varepsilon (t_{1} , u (t_{1} ))) \vert {>} \eta _{1} \vert u \vert + \kappa , \vert u \vert {>} d , \end{aligned}$$

leads to the inequality

$$\begin{aligned} \vert u (t_{1} - \varepsilon (t_{1} , u (t_{1} ))) \vert \le d . \end{aligned}$$

**Case 2.** Let $\eta _{1} {>} 0$. If $\vert u (t_{1} - \varepsilon (t_{1} , u (t_{1} ))) \vert {>} d ,$ then it arrives at the conclusion of (5) and the hypotheses (H2) and (H3),

$$\begin{aligned} \begin{aligned} \eta _{1} \vert u (t_{1} - \varepsilon (t_{1} , u (t_{1} ))) \vert + \kappa&< \vert \vartheta (t_{1} , u ( t_{1} - \varepsilon _{1} (t_{1} , u ))) \vert \\&\le \eta _{1} \vert D^{\mu } u ( t_{1} - \varpi ) \vert + \kappa . \end{aligned} \end{aligned}$$

From the above inequality, we conclude that

$$\begin{aligned} \begin{aligned} \vert u (t_{1} - \varepsilon (t_{1} , u (t_{1} )))\vert&\le \vert D^{\mu } u (t_{1} - \varpi )\vert \le \Vert D^{\mu } u \Vert _{0}\\&\le \Vert D^{\mu } u \Vert _{0} + d. \end{aligned} \end{aligned}$$

We note that *u*(*t*) is periodic and there exists a $\overline{t} \in [ 0,2 \pi ]$ such that

$$\begin{aligned} \vert u (\overline{t} ) \vert \le \Vert D^{\mu } u \Vert _{0} + d \end{aligned}$$

holds. Using Lemma 1, for all $\overline{t} \in ( 0, \infty ),$ we have

$$\begin{aligned}\Vert u \Vert _{0} &\le \vert u ( \overline{t} )\vert + \frac{1}{2} \int ^{2 \pi } _{0} \vert D^{\mu } u ( \delta ) \vert d \delta \\ &\le ( \pi + 1 )\Vert D^{\mu } u\Vert _{0} + d\\ \Rightarrow \Vert D^{\mu } u \Vert _{0} &\le (\pi + 1 ) \Vert D^{\mu } u \Vert _{0} + d . \end{aligned}$$

Hence, for all $t_{\bullet } \in [0 , \infty ),$ we obtain

$$\begin{aligned} \Vert D^{\mu } u \Vert _{0} \le \vert D^{\mu } u ( t_{\bullet } )\vert + \frac{1}{2} \int ^{2 \pi } _{0} \vert D^{2 \mu } u ( \delta ) \vert d\delta . \end{aligned}$$

Because of $u(0)=u( 2 \pi )$, then it follows from the mean-value theorem for the Riemann–Liouville fractional derivative (see Abramovich et al. 2010) that there exists $t_{\bullet }:= \varrho \in [ 0,2 \pi )$ such that $D^\mu u(\varrho )=0.$ Thus, we have

$$\begin{aligned} \Vert D^{\mu } u \Vert _{0} \le \frac{1}{2} \int ^{2 \pi } _{0} \vert D^{2\mu } u (\delta )\vert d \delta . \end{aligned}$$

Let

$$\begin{aligned} \Xi _{1} &= \lbrace t : t \in [0 , 2\pi ] , u(t - \varepsilon (t , u (t))) {{>}} d \rbrace ,\\ \Xi _{2} &= \lbrace t : t \in [0 , 2\pi ] , u(t - \varepsilon (t , u (t))) {<} -d \rbrace , \end{aligned}$$

and

$$\begin{aligned} \Xi _{3} = \lbrace t : t \in [0 , 2\pi ] , u(t - \varepsilon (t , u (t))) \le d \rbrace . \end{aligned}$$

In virtue of the Eq. (4), we conclude that

$$\begin{aligned}&\int _{\Xi _{1}} \vert \vartheta ( \delta , u (\delta - \varepsilon ( \delta , u (u))))\vert d \delta \le \int ^{2\pi } _{0} \vert \varphi (\delta , D^{\mu } u (\delta - \varpi )) \vert d \delta \\&\quad +\left( \int _{\Xi _{2}} +\int _{\Xi _{3}} \right) \vert \vartheta (\delta , u ( \delta - \varepsilon (\delta , u (\delta ))))\vert d \delta . \end{aligned}$$

Consequently, we attain

$$\begin{aligned}&\Vert D^{\mu } u \Vert _{0} \le \frac{1}{2} \int ^{2\pi } _{0} \vert D^{2\mu } u (\delta )\vert d\delta \le \frac{1}{2} \int ^{2\pi } _{0} \vert \varphi (\delta , D^{\mu } u ( \delta - \varpi ))\vert d\delta +\frac{1}{2} \int _{0}^{2\pi } \vert \Psi (u(\delta ))D^{\mu } u (\delta )\vert d\delta \\&\qquad +\frac{1}{2} \int _{0}^{2\pi } \vert \vartheta (\delta , u ( \delta - \varepsilon (\delta , u (\delta ))))\vert d\delta + \frac{1}{2} \int ^{2\pi }_{0} \vert p ( \delta ) \vert d\delta \le \frac{1}{2} \int ^{2\pi }_{0} \vert \varphi (\delta , D^{\mu } u ( \delta - \varpi ))\vert d\delta \\&\qquad +\frac{1}{2} \int _{0}^{2\pi } \vert \Psi (u(\delta )) D^{\mu } u (\delta ) \vert d\delta + \frac{1}{2} \left( \int _{\Xi _{1}} +\int _{\Xi _{2}}+ \int _{\Xi _{3}} \right) \vert \vartheta (\delta ,u (\delta -\varepsilon (\delta , u (\delta )))\vert d\delta + \pi \Vert p \Vert _{0} \\&\quad \le \int ^{2\pi }_{0} \vert \varphi (\delta , D^{\mu } u ( \delta - \varpi ))\vert d\delta + \Upsilon _{1} \pi \Vert D^{\mu } u\Vert _{0} \\&\qquad + \left( \int _{\Xi _{2}} + \int _{\Xi _{3}} \right) \vert \vartheta (\delta ,u (\delta -\varepsilon (\delta , u (\delta )))\vert d\delta + \pi \Vert p \Vert _{0} \\&\quad \le 2 \pi \lbrace ( \Upsilon _{1} + \eta _{1} )\Vert D^{\mu } u \Vert _{0} + \eta _{2} \Vert u \Vert _{0} \rbrace + 2\pi (\kappa + m + \vartheta _{d} ) + \pi \Vert p \Vert _{0} \\&\quad \le 2 \pi \lbrace ( \Upsilon _{1} + \eta _{1} )+ ( \pi + 1 ) \eta _{2} \Vert D^{\mu } u \Vert _{0} \rbrace \\&\qquad + 2\pi (\kappa + m + \vartheta _{d} + \eta _{2} d ) + \pi \Vert p \Vert _{0}\\&\quad \Rightarrow \Vert D^{\mu } u \Vert _{0} \le 2 \pi \lbrace ( \Upsilon _{1} + \eta _{1} )+ ( \pi + 1 ) \eta _{2} \Vert \rbrace D^{\mu } u \Vert _{0} + 2\pi (\kappa + m + \vartheta _{d} + \eta _{2} d ) + \pi \Vert p \Vert _{0} \\&\quad \Rightarrow \Vert D^{\mu } u \Vert _{0} \le \frac{2\pi (\kappa + m + \vartheta _{d} + \eta _{2} d ) + \pi \Vert p \Vert _{0}}{1 - 2 \pi \lbrace ( \Upsilon _{1} + \eta _{1} )+ ( \pi + 1 ) \eta _{2}\rbrace } = m_{1} , \end{aligned}$$

where

$$\begin{aligned} \vartheta _{d} = max_{t\in [0,2\pi ] , \vert u \vert \le d} \vert \vartheta ( t , u ) \vert . \end{aligned}$$

By the inequality

$$\begin{aligned} \Vert u \Vert _{0} \le (\pi + 1) \Vert D^{\mu } u \Vert _{0} + d, \end{aligned}$$

one can calculate that

$$\begin{aligned} \Vert u \Vert _{0} \le (\pi + 1) m_{1} + d. \end{aligned}$$

This completes the proof of Theorem 1.

Theorem 1 shows that the solution is bounded by its fractional derivative in a fractional space. Therefore, Lemma 1 and Theorem 1 imply the periodicity of the solution in a bounded domain. Our next result illustrates different types of assumptions to get the periodicity and positivity of Eq. (2) and hence Eq. (1).

### **Theorem 2**

*Assume that there exist constants*

$$\begin{aligned} \Big (\Upsilon _{2} {>} 0, \, \eta _{1}, \, \eta _{2} \ge 0, 0< d < \infty , \kappa {>} 0\,\, \text {and} \,\, m {>} 0\Big ) \end{aligned}$$

*such that the following conditions hold:*

1.   $$\vert \Psi (u) \vert \le \Upsilon _{2}$$ for all $$u \in \mathbb {R} ,$$
2.   $$\vert \varphi (t,u) \vert \le \Upsilon _{2} u + \kappa$$ for all $$(t ,u) \in J \times \mathbb {R} ,$$
3.
   
    $u \vartheta (t ,u (t-\varepsilon (t,u ))) {>} 0;$ this implies that $\vartheta {>} 0$
    $$\vartheta (t , u (t - \varepsilon (t, u))) {>} \eta _{1} u + \kappa$$ for all $$t \in J , u {>} d ,$$
4.   $$\vartheta (t ,u (t-\varepsilon (t,u ))) {>} \eta _{2} u - m$$ for all $$t \in J , u \ge d .$$

*If*

$$\begin{aligned} 2 \pi \lbrace (\Upsilon _{2} + \eta _{1} ) + (\pi + 1 ) \eta _{2} \rbrace < 1 , \end{aligned}$$

*then Eq.* (1) *has at least one*$2 \pi$-*periodic solution.*

### *Proof*

We again consider the auxiliary equation, Eq. (2). Let *u*(*t*) be any $2 \pi -$periodic solution of Eq. (2). By the hypothesis (H3) and (H4), we conclude that there exists $t_\bullet \in J$ and a positive constant $d_\bullet ,$ such that

$$\begin{aligned} d_\bullet = u(t_\bullet ) {>} d. \end{aligned}$$

In this case, we define the following sets:

$$\begin{aligned} {\$}_{1}&= \lbrace t : t \in [0 , 2\pi ] , u(t - \varepsilon (t , u (t))) {{\ge }} d \rbrace ,\\ {\$}_{2}&= \lbrace t : t \in [0 , 2\pi ] , u(t - \varepsilon (t , u (t))) {{\le }} \overline{d}, \, \overline{d} {{\ge }} \max d, \rbrace . \end{aligned}$$

and

$$\begin{aligned} \$_{3} = \lbrace t : t \in [0 , 2\pi ] , u(t - \varepsilon (t , u (t))) = d_\bullet \rbrace . \end{aligned}$$

A calculation implies that

$$\begin{aligned} \int _{\$_{1}} \vartheta ( \delta , u (\delta - \varepsilon ( \delta , u (u))))\, d \delta \le \int ^{2\pi } _{0} \vert \varphi (\delta , D^{\mu } u (\delta - \varpi )) \vert d \delta +\left( \int _{\$_{2}}+ \int _{\$_{3}} \right) \vartheta (\delta , u ( \delta - \varepsilon (\delta , u (\delta ))))\, d \delta . \end{aligned}$$

Moreover, we have

$$\begin{aligned}&\Vert D^{\mu } u \Vert _{0} \le \frac{1}{2} \int ^{2\pi } _{0} \vert D^{2\mu } u (\delta )\vert d\delta \le \frac{1}{2} \int ^{2\pi } _{0} \vert \varphi (\delta , D^{\mu } u ( \delta - \varpi ))\vert d\delta +\frac{1}{2} \int _{0}^{2\pi } \vert \Psi (u(\delta ))D^{\mu } u (\delta )\vert d\delta \\&\qquad +\frac{1}{2} \int _{0}^{2\pi } \vartheta (\delta , u ( \delta - \varepsilon (\delta , u (\delta ))))\, d\delta + \frac{1}{2} \int ^{2\pi }_{0} \vert p ( \delta ) \vert d\delta \le \frac{1}{2} \int ^{2\pi }_{0} \vert \varphi (\delta , D^{\mu } u ( \delta - \varpi ))\vert d\delta \\&\qquad +\frac{1}{2} \int _{0}^{2\pi } \vert \Psi (u(\delta )) D^{\mu } u (\delta ) \vert d\delta + \frac{1}{2} \left( \int _{\$_{1}}+ \int _{\$_{2}}+ \int _{\$_{3}} \right) \vartheta (\delta ,u (\delta -\varepsilon (\delta , u (\delta )))\, d\delta + \pi \Vert p \Vert _{0}\\&\quad \le \int ^{2\pi }_{0} \vert \varphi (\delta , D^{\mu } u ( \delta - \varpi ))\vert d\delta + \Upsilon _{1} \pi \Vert D^{\mu } u\Vert _{0}\\&\qquad + \left( \int _{\$_{2}} +\int _{\$_{3}} \right) \vartheta (\delta ,u (\delta -\varepsilon (\delta , u (\delta )))\, d\delta + \pi \Vert p \Vert _{0}\\&\quad \le 2 \pi \lbrace ( \Upsilon _{2} + \eta _{1} )\Vert D^{\mu } u \Vert _{0} + \eta _{2} \Vert u \Vert _{0} \rbrace + 2\pi (\kappa + m + \vartheta _{d} ) + \pi \Vert p \Vert _{0}\\&\quad \le 2 \pi \lbrace ( \Upsilon _{2} + \eta _{1} )+ ( \pi + 1 ) \eta _{2} \Vert D^{\mu } u \Vert _{0} \rbrace \\&\qquad + 2\pi (\kappa + m + \vartheta _{d} + \eta _{2} d ) + \pi \Vert p \Vert _{0}\\&\quad \Rightarrow \Vert D^{\mu } u \Vert _{0} \le 2 \pi \lbrace ( \Upsilon _{2} + \eta _{1} )+ ( \pi + 1 ) \eta _{2} \Vert \rbrace D^{\mu } u \Vert _{0} + 2\pi (\kappa + m + \vartheta _{d} + \eta _{2} d ) + \pi \Vert p \Vert _{0}\\&\quad \Rightarrow \Vert D^{\mu } u \Vert _{0} \le \frac{2\pi (\kappa + m + \vartheta _{d} + \eta _{2} d ) + \pi \Vert p \Vert _{0}}{1 - 2 \pi \lbrace ( \Upsilon _{2} + \eta _{1} )+ ( \pi + 1 ) \eta _{2}\rbrace } = m_{2}. \end{aligned}$$

By the inequality

$$\begin{aligned} \Vert u \Vert _{0} \le (\pi + 1) \Vert D^{\mu } u \Vert _{0} + d \end{aligned}$$

one can have

$$\begin{aligned} \Vert u \Vert _{0} \le (\pi + 1) m_{2} + d \end{aligned}$$

This completes the proof of Theorem 2.

It is clear that the solution in Theorem 2 is positive as well as periodic.

### *Remark 1*

When $\mu =1, \, \Psi (u)\equiv 0$ and $\varepsilon (t, u (t)) \equiv \varepsilon (t),$ (constant) and $\eta _{1} = \eta _{2} = 0,$ then the conditions of Theorems 1 and Theorem 2 reduce to the outcomes in Zhou and Tang (2007), (see Theorems 2.1, 2.2), respectively. Therefore, our results generalize and improve the corresponding results in Zhou and Tang (2007).

### *Remark 2*

When   $\mu =1,\, \Psi (u)\equiv 0$ and $\varepsilon (t, u (t)) \equiv \varepsilon (t),$ then the conditions of Theorem 1 and Theorem 2 yield the outcomes in Wang and Yan (2000)   (see Theorems 2.1, 2.2), respectively. Therefore, our results generalize and improve the results in Wang and Yan (2000).

### *Remark 3*

When  $\mu =1,$ we obtain the result that given in Tunç (2014).

### *Example 1*

Suppose the equation

6 $$\begin{aligned} D^{2 \mu }u(t) =\,\, - \frac{1}{\ell } D^{ \mu }u(t)- u(t) - \frac{1}{\ell } \exp (- \varepsilon (t,u))\cos (t), \end{aligned}$$

where

$$\begin{aligned} \Big (\Psi =\frac{1}{\ell }, \, \varphi (t)=u(t), \, \vartheta (t,u)= \frac{1}{\ell } \exp (- \varepsilon (t,u))\cos (t), \, p(t)=0,\Big ) \end{aligned}$$

such that

$$\begin{aligned} \Big (\varepsilon (t,u)=\frac{t}{\ell }, \, t \in [0,1] \,\, \text {and} \,\,\ell {>} \frac{1}{2\pi -1} \Big ). \end{aligned}$$

Subjected to the initial condition

$$\begin{aligned} \Big ( u(0) =0, \,\, u'(0)=1 \Big ). \end{aligned}$$

Thus, we have $|\Psi | = \frac{1}{\ell } : = \Upsilon _1, \, |\varphi | \le \Upsilon _1 |u|, \, \vartheta (t,u)\in [0,4 \pi ]$ for $\, t \in [0,2 \pi ]$ with $\eta _1=1, \eta _2=0$ and $m=0.$ Consequently the condition of Theorem 1 is satisfied i.e $\frac{1}{\ell } 2 \pi -1.$ Hence, Eq. (6) has a periodic solution. Note that for $\mu =1,$ the equation has the exact solution takes the form

$$\begin{aligned} u(t)= \exp \left(- \frac{t}{\ell }\right) \sin (t), \quad t \ge 0. \end{aligned}$$

Moreover, when $\ell =5$ and $\varepsilon (t,u)=t,$ we have a result given in Nemati et al. (2014).

## Conclusions

In general, we know that the Riemann–Liouville fractional operator is not periodic. In this effort, we introduced a construction to get the periodicity of some classes of fractional differential equations. A Rayleigh-type equation with state-dependent delay was considered in this occasion. The existence of periodic solutions to this equation was investigated. We utilized Riemann–Liouville fractional derivatives during the generalization, we obtained sufficient conditions for the existence of periodic solutions. Moreover, we have extended and improved some results from the recent literatures.

## References

[CR1] Abramovich S, Farid G, Pecaric J, Dragomir SS (2010). More about Hermite-Hadamard inequalities, Cauchy’s means, and superquadracity. J Inequal Appl.

[CR2] Agarwal R, Andrade B, Cuevas C (2010). On type of periodicity and ergodicity to a class of fractional order differential equations. Adv Diff Equ.

[CR3] Gaines RE, Mawhin JL (1977) Coincidence degree, and nonlinear differential equations

[CR4] Gorenflo R, Mainardi F (2008) Fractional calculus: integral and differential equations of fractional order. arXiv preprint arXiv:0805.3823

[CR5] Ibrahim RW, Jahangiri JM (2015). Existence of fractional differential chains and factorizations based on transformations. Math Methods Appl Sci.

[CR6] Khorshidi M, Nadjafikhah M, Jafari H (2015) Fractional derivative generalization of Noethers theorem. Open Math 13(1)

[CR7] Kilbas A Anatolii Aleksandrovich, Srivastava Hari Mohan, Trujillo Juan J (2006). Theory and applications of fractional differential equations.

[CR8] Liu Fawang (2010). Fractional differential equations. Int J Differ Equ.

[CR9] Nemati K, Shamsuddin SM, Darus M (2014). An optimization technique based on imperialist competition algorithm to measurement of error for solving initial and boundary value problems. Measurement.

[CR10] Podlubny I (1999). Fractional differential equations.

[CR11] Rakkiyappan R, Sivaranjani R, Velmurugan G, Cao J (2016) Analysis of global $$O (t- a)$$ stability and global asymptotical periodicity for a class of fractional-order complex-valued neural networks with time varying delays. Neural Netw 10.1016/j.neunet.2016.01.007 26922720

[CR12] Strutt JW (1877) [Lord Rayleigh] Theory of sound, 1, Dover Publications, New York (re-issued 1945)

[CR13] Tarasov V (2010). Fractional dynamics: applications of fractional calculus to dynamics of particles, fields and media.

[CR14] Tunç C (2014). New results on the existence of periodic solutions for Rayleigh equation with state-dependent delay. J Math Fundam Sci.

[CR15] Wang G, Yan J (2000). On existence of periodic solutions of the Rayleigh equation of retarded type. Int J Math Math Sci.

[CR16] Wang Y, Zhang L (2009). Existence of asymptotically stable periodic solutions of a Rayleigh type equation. Nonlinear Anal Theory Methods Appl.

[CR17] Yang X-J (2012). Advanced local fractional calculus and its applications.

[CR18] Yang X-J (2015). Local fractional integral transforms and their applications.

[CR19] Zhou Y, Tang X (2007). On existence of periodic solutions of Rayleigh equation of retarded type. J Comput Appl Math.

